# *In silico* Analysis Revealed Potential Anti-SARS-CoV-2 Main Protease Activity by the Zonulin Inhibitor Larazotide Acetate

**DOI:** 10.3389/fchem.2020.628609

**Published:** 2021-01-15

**Authors:** Simone Di Micco, Simona Musella, Maria C. Scala, Marina Sala, Pietro Campiglia, Giuseppe Bifulco, Alessio Fasano

**Affiliations:** ^1^European Biomedical Research Institute of Salerno (EBRIS), Salerno, Italy; ^2^Department of Pharmacy, University of Salerno, Salerno, Italy; ^3^Mucosal Immunology and Biology Research Center, Massachusetts General Hospital–Harvard Medical School, Boston, MA, United States

**Keywords:** M^pro^ inhibitor, molecular docking, molecular dynamics, MM-GBSA, drug repurposing

## Abstract

The most severe outcome of COVID-19 infection is the development of interstitial pneumonia causing acute lung injury (ALI) and/or acute respiratory distress syndrome (ARDS), both responsible for the infected patients' mortality. ALI and ARDS are characterized by a leakage of plasma components into the lungs, compromising their ability to expand and optimally engage in gas exchange with blood, resulting in respiratory failure. We have previously reported that zonulin, a protein dictating epithelial and endothelial permeability in several districts, including the airways, is involved in ALI pathogenesis in mouse models, and that its peptide inhibitor Larazotide acetate (also called AT1001) ameliorated ALI and subsequent mortality by decreasing mucosal permeability to fluid and extravasation of neutrophils into the lungs. With the recent crystallographic resolution of the SARS-CoV-2 main protease (M^pro^), an enzyme fundamental in the viral lifecycle, bound to peptidomimetic inhibitors N3 and 13b, we were able to perform molecular modeling investigation showing that AT1001 presents structural motifs similar to co-crystallized ligands. Specifically, molecular docking, MM-GBSA-based predictions and molecular dynamics showed that AT1001 docks extremely well in the M^pro^ catalytic domain through a global turn conformational arrangement without any unfavorable steric hindrance. Finally, we have observed that AT1001 can be superimposed onto the crystallized structures of N3 and 13b, establishing a higher number of interactions and accordingly a tighter binding. *In vitro* studies confirmed AT1001 anti-M^pro^ and preliminary investigation indicted an anti-viral activity. Combined, these studies suggest that AT1001, besides its well-demonstrated effect in ameliorating mucosal permeability in ALI/ARDS, may also exert a direct anti-SARS-CoV-2 effect by blocking the M^pro^. AT1001 has been used extensively in a variety of animal models of ALI demonstrating robust safety and efficacy; it is currently in phase 3 trials in celiac subjects showing strong safety and efficacy profiles. We therefore propose its use as a specific anti-SARS-CoV-2 multitargeting treatment for the current pandemic.

## Introduction

COVID-19 is a serious and potentially life-threatening disease triggered by the SARS-CoV-2 virus. To date, the COVID-19 pandemic has infected ~73 million people around the world and claimed nearly 1.7 million lives. As described above, patients with severe cases of COVID-19 experience life-threatening viral pneumonia, which can advance to ALI, and/or ARDS and then death. While the clinical course of patients infected with COVID-19 has not been fully characterized, severe cases involve acute, and fatal respiratory symptoms. Available treatment for ALI/ARDS in this patient population is limited and there is currently no proven and effective cure that has been approved for treatment of SARS-CoV-2 infection. ARDS is an acute, life-threatening inflammatory lung injury exhibiting a lack of oxygen to the tissue, hypoxia, and stiff lungs induced by an increased pulmonary vascular permeability (Ranieri et al., 2012). ARDS necessitates hospitalization and mechanical ventilation. Given the limited number of hospital beds and ventilators worldwide, a fast increment of ARDS patients constitutes the major problem for the global public health system. When implementing standard of care, including mechanical ventilation, ARDS has an overall mortality rate of 88% (Richardson et al., 2020). A recent retrospective study of COVID-19 patients with ARDS in China (Zhou et al., 2020) concluded that, among the 109 patients studied, those with moderate and severe ARDS had higher mortality rates, and no significant effect of antiviral, glucocorticoid, or immunoglobulin treatment on survival was observed. ALI and ARDS cause a plasma component leakage into the lungs, affecting their ability to expand and optimally exchange gas with blood, giving rise to respiratory failure (Ware and Matthay, 2000). ALI/ARDS can be originated by several insults, and the consequent pathophysiology results highly complex. Even though the original processes inflammatory response into the lung may diverge for the causative stimulus, the downstream happenings commonly evolve into a ALI feature of increased lung vascular permeability (Huber-Lang et al., 2006). In ALI pathogenesis, local activation of the complement system is crucial for the launch and progression of illness, and products of complement activation can stimulate the oxygen radical release, the adhesion molecule yield on both endothelial cells and leukocytes, and the expression of cytokines and chemokines. Moreover, the complement activation product C5a can be a direct chemoattractant for neutrophils. Despite the vast body of experimental and clinical investigation into the immunopathogenesis of ALI, the mechanisms leading to a loss of endothelial and epithelial barriers in the lung are still poorly understood. Our discovery of zonulin (Wang et al., 2000), a molecule that regulates epithelial and endothelial permeability in several districts including the airways, shed some light on these mechanisms. Zonulin is a member of a family of related proteins (Fasano, 2011; Sturgeon and Fasano, 2016; Valitutti and Fasano, 2019) whose first member, pre-haptoglobin 2 (HP2), was discovered almost ten years ago (Tripathi et al., 2009). Due to mutations in the catalytic domain, haptoglobins lost their protease function through evolution and subsequently acquired new biological roles. These include modulating intercellular tight junctions (TJs) (Richardson et al., 2020), which are structures that control the epithelial and endothelial paracellular trafficking of fluid and molecules. Along with this high mutation rate, frequent zonulin polymorphisms gave rise to a family of structurally and functionally related zonulins during evolution, such as properdin and pre-HP2 (Scheffler et al., 2018).

The inappropriate production of increased amounts of zonulin triggered by several stimuli, including viral infections, provokes a barrier function loss, entailing an uncontrolled antigen trafficking, initiating an intrinsic immune response by the submucosal compartment. By continuing this process, the consequent adaptive immune response triggers the production of pro-inflammatory cytokines (tumor necrosis factor alpha [TNF α] and interferon gamma [IFN-γ]), which causes further opening of the paracellular route for antigens. Finally, these originated mechanisms induce a tolerance break with a subsequent inflammation. The nature of the resultant inflammation is influenced by the genetic background of the specific host, which dictates what organ or tissue will be targeted by the inflammatory process. Because of zonulin's involvement in a multitude of inflammatory diseases, including ALI and pneumonia, zonulin's inhibitor, AT1001, a synthetic octapeptide zonulin receptor antagonist (Di Pierro et al., 2001) has been investigated extensively in several animal models of inflammation showing strong efficacy data. AT1001 is currently in Phase 3 trials (with the name Larazotide acetate) in subjects affected by celiac disease and shows strong safety and efficacy profiles for this indication.

Due to the high diffusion rate of SARS-CoV-2 infection and the high frequency of complications due to ALI or ARDS, there is an incumbent risk of a high number of patients requiring hospitalization and subsequent assisted ventilation that may exceed the capacity of public health care systems to provide adequate assistance. To date, there are no proven therapies available to effectively treat COVID-19 infection. The discovery of a cure is urgently warranted but a potential breakthrough is limited by the time-consuming process traditionally needed to develop new drugs or vaccines.

## Materials and Methods

### Molecular Docking

The 3D structure of the **2**–M^pro^ was constructed through Build Panel of Maestro (version 11), and its geometry was optimized by applying: Polak-Ribière conjugate gradient algorithm (maximum derivative <0.001 kcal/mol), OPLS3 force field (Harder et al., 2016), and GB/SA (generalized Born/surface area) (Still et al., 1990) solvent treatment to mimic the H_2_O solvation. The tested ligands were then processed by LigPrep, considering the protonation states at pH of 7.0 ± 1.0. The X-ray structure of M^pro^ (PDB ID: 6UL7) (Jin et al., 2020) was used as macromolecular model and managed by Protein Preparation Wizard (Sastry et al., 2013; Protein Preparation Wizard Schrödinger LLC, 2017): bond order assignment, hydrogen addition; missing side chain and loop check; check of alternate positions of the residues. The side chain charges were given considering their pK_a_ at pH 7.0 ± 1.0. The network of hydrogen bonds was enhanced through the optimize preference. The H_2_O molecules were removed. Glide (Tubert-Brohman et al., 2013) was used for molecular docking calculations. A receptor grid suitable for peptide docking was generated with inner and outer boxes of 10 and 22 Å, respectively, and centered on x, y, and z coordinates: −10.80, 12.53, 68.70. A first round of SP-peptide (Standard Precision) (Tubert-Brohman et al., 2013) was used, applying default parameters with extended sampling option, generating 100 poses for **2** treated as flexible. The docked poses generated from the first round were used as input conformations for a second run with the same parameters. All conformations from the first two rounds were ranked by docking score, and the best 100 conformers were used as input for a third calculation run. Ring conformation sampling was accomplished by applying an energy cuttoff of 2.5 kcal/mol. The enhanced sampling mode was utilized, retaining 10,0000 poses/ligand for docking initial step, picking 1,000 poses per ligand in the energy minimization. The value of 0.8 was applied as the scaling factor for van der Waals radii and 0.15 as the partial charge cutoff. On docking outputs, a postdocking optimization step was run, considering 100 maximum poses and applying a cutoff of 0.5 kcal/mol. Epik state penalty; reward of intramolecular H-bonds; and aromatic hydrogen were taken into account. Tideglusib was flexibly docked by one run of Standard Precision (SP) followed by three rounds of Extra Precision (XP) Glide mode step (Di Micco et al., 2018; Gerstmeier et al., 2019). By the SP round of docking calculations, ten poses were generated and employed as initial conformers in the XP predictions. Nitrogen inversion and ring conformers (with 2.5 kcal/mol energetic cutoff) were sampled in all calculations. For SP run, the enhanced sampling mode was used, maintaining 5,000 poses/ligand in docking initial step, choosing 400 poses/ligand for energetic refinement. In the XP calculations, the enhanced sampling mode was applied maintaining 10,000 poses/ligand in the initial docking step, sorting 1,000 poses/ligand for energetic refinement. One thousand maximum output conformations were saved, by applying 0.15 and 0.8 as partial charge cutoff and scaling factor for van der Waals radii, respectively. Docking outputs were optimized, selecting 1,000 poses, and by using 0.5 kcal/mol limit. Epik state penalty; reward of intramolecular H-bonds; aromatic H-bonds were taken into account as energy contributions. The energetic cutoff of 2.5 kcal/mol was used for ring conformation sampling; penalizing structures endowed with non-planar peptide bond. The docked poses were ranked by docking score. Maestro (version 11) was utilized for theoretic study and to generate all depictions.

### MM-GBSA

MM-GBSA predictions were achieved by the Prime (Knight et al., 2014) module of the Schrödinger suite with default parameters. A distance of 5 Å from the ligand was used to define flexible residues. For alanine scanning, the receptor was fixed.

### Molecular Dynamics

The docked complex **2**-M^pro^ was used for molecular dynamics simulation. The complex was prepared by System Builder (System Builder, 2015) in Desmond (Bowers et al., 2006; Desmond, DE Shaw Research, 2017). A cubic box with a 10 Å buffer distance was employed, obtaining a 56677 atom-system. The OPLS3 force field and the TIP3P (Jorgensen et al., 1983) solvation model were applied. For electroneutrality, Na^+^ and Cl^−^ ions were included. An additional NaCl solution (0.15 M) was applied. This system was optimized through the LBFGS methodology, using maximum 2,000 iterations and 1.0 kcal/mol/Å as convergence threshold. On the minimized molecular system, the following relaxation protocol was employed: (1) NVT simulation (2 ns, 10 K), by small time steps and restraining solute heavy atoms; (2) NVT simulation (240 ps, 10 K with Berendsen thermostat), using fast temperature relaxation constant, velocity resampling every 1 ps, and restraining solute heavy atoms; (3) NPT simulation (240 ps) by means of Berendsen thermostat and Berendsen barostat (10 K), 1 atm pressure, fast temperature relaxation constant, slow pressure relaxation constant, 1 ps for velocity resampling, restraining heavy solute atoms; (4) NPT ensemble simulation (240 ps) by means of Berendsen thermostat (310 K) and a Berendsen barostat (1 atm), using fast temperature relaxation constant, a slow pressure relaxation constant, 1 ps for velocity resampling, restraining heavy solute atoms; (5) NPT simulation (480 ps) by Berendsen thermostat (310 K) and a Berendsen barostat (1 atm), using fast temperature relaxation constant and normal pressure relaxation constant. Two replicas of molecular dynamics simulation of 100 ns (310 K) were done, by recording each 1.2 ps and through NPT (1.01 bar) ensemble class. A 2.0 fs integration time step was applied. The Simulation Quality Analysis tool of Desmond was used to evaluate every equilibration phase, inspecting: total and potential energies, temperature, pressure and volume.

### Material and Chemicals

Coupling agents (HOAt, HBTU), Fmoc-Gly-Wang, Nα-Fmoc-protected amino acids, DIEA, piperidine, and trifluoroacetic acid were supplied by Iris Biotech (Marktredwitz, Germany). Remaining reagents and solvents were of analytical grade, commercially available, and were utilized without any further purification.

### Peptide Synthesis

The synthesis of AT1001 was carried out by a solid phase methodology by means of standard Fmoc method on a Biotage Initiator + Alstra automated microwave synthesizer (Biotage, Uppsala, Sweden). Peptide was manufactured on an Fmoc-L-Ile-Wang resin (0.7 mmol/g, 150 mg), formerly deprotected by 25% of piperidine/DMF (1 × 3 min, 1 × 10 min) at room temperature. Subsequently, the resin was rinsed by DMF (4 × 4.5 ml) and protected amino acids attached to the resin stepwise. Coupling reactions were accomplished by means of Nα-Fmoc amino acids (4.0 eq., 0.5 M), HBTU (3eq, 0.6 M), HOAt (3eq, 0.5 M), and DIEA (6eq, 2 M) in N-methyl-2- pyrrolidone (NMP) for 10 min at 75 °C (2×). Following every coupling step, the Fmoc protecting group was removed as described above. The resin was rinsed using DMF (4 × 4.5 ml) after each coupling and deprotection stage. The N-terminal Fmoc group was separated, the resin was rinsed using DCM (7x), and the synthesized molecule released from the resin by using TFA/TIS/H_2_O (ratio 90:5:5) for 3 h. The resin was separated by filtration and the crude peptide recuperated by precipitation by means of cold anhydrous ethyl ether obtaining a white powder that was then lyophilized.

### Purification and Characterization

Finally, crude peptide was purified by RP-HPLC employing a preparative C18-bonded silica column (Phenomenex, AXIA Kinetex 5 μm 100 Å, 100 × 21.20 mm) by mean of a Shimadzu SPD 10A UV/vis detector, through detection at 210 and 254 nm. Flow rate of 17 mL/min with solvent A (10%, v/v, water in 0.1% aqueous TFA), and a linear gradient from 10 to 90% of solvent B (80%, v/v, acetonitrile in 0.1% aqueous TFA) over 30 min was used for peptide elution. Analytical purity and retention time (tr) of AT1001 was established by means of the same solvents A and B with a flow rate of 0.5 mL/min and a linear gradient from 10 to 90% B over 15 min, fitted with analytical C-18 column (Phenomenex, Aeris XB-C18 column, 100 mm × 2.1, 3.6 μm). Peptide presented >99% purity, checked at 215 nm. Molecular weight was determined by FT MS+pESI full spectrometry (LTQ Orbitrap XL, Thermo Scientific). Analytical data are shown in Supplementary Materials (Supplementary Figures 3, 4).

### Enzymatic Inhibition Assays

The recombinant SARS-CoV-2 M^pro^ (Proteros) (20 nM at a final concentration) was mixed with serial dilutions of AT1001 and Dabcyl-KTSAVLQSGFRKM-E(Edans)-NH_2_ substrate (5 μM) in 20 μL (reaction volume) assay buffer solution (20 mM HEPES, pH 7.5, 1 mM DTT, 1 mM EDTA, 100 mM NaCl, 0.01% Tween20). Fluorescence signal was monitored every 30 s for 10 min. 18 concentrations of AT1001 and three independent experiments were performed at room temperature. The fluorescence signal of the Edans was monitored at an emission wavelength of 500 nm by exciting at 360 nm, by means of Pherastar FSX microplate Reader. Calpeptin was used as reference to set up the experiments. All experimental data was analyzed using GraphPad Prism software.

## Results

**Figure 1:**
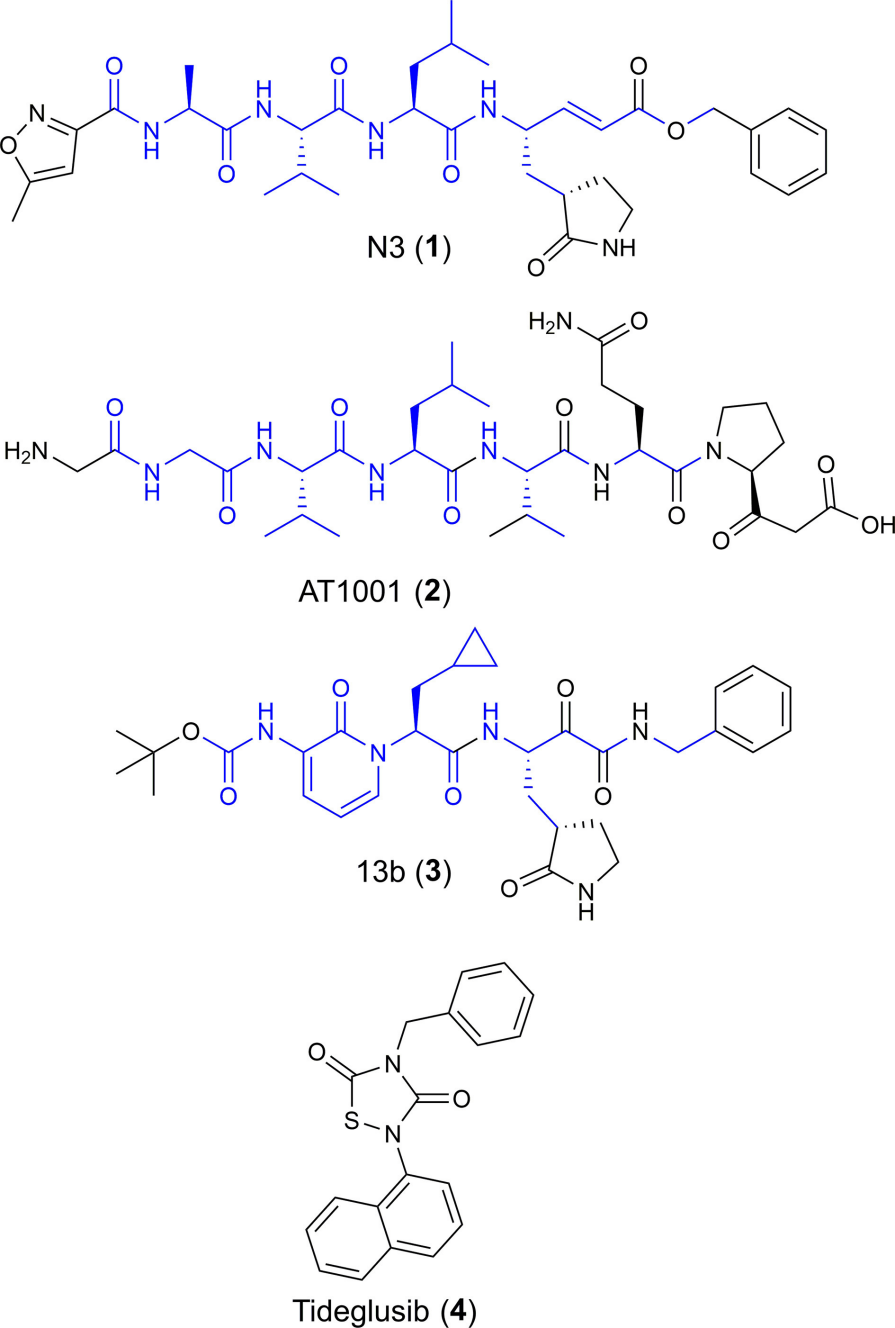
Chemical structures of four potential M^pro^ inhibitors, namely N3 (**1**), AT1001 (**2**), 13b (**3**) and Tideglusib (**4**). The blue portions indicate the similar structural motifs of AT1001 compared to N3 and 13b.

Based on the aforementioned considerations, we envisaged drug repurposing (Giordano et al., 2018a) as an appropriate strategy to provide a fast response to COVID-19 pandemic. Recently, Yang et al. reported the first X-ray structure of the main protease of the COVID-19 virus (M^pro^, PDB ID: 6L U7) (Jin et al., 2020) covalently bound to N3 (Figure 1), a peptide-like inhibitor acting in low micromolar range. This enzyme, also named as 3C-like protease, is a cysteine protease of 33.8-kDa, which plays a key role in the digestion of two overlapping (pp1a and pp1ab) polyproteins that are translated from the viral RNA (Hilgenfeld, 2014). These polyproteins are fundamental for viral transcription and replication (Anand et al., 2002; Yang et al., 2003).

The M^pro^ acts at no <11 cleavage sites (↓) on its polymer substrate and mostly recognizes the sequence: Leu-Gln↓(Ser,Ala,Gly). Thus, blocking the M^pro^ activity would prevent viral replication. This enzyme is highly attractive as an antiviral drug target. It is instrumental for replication of the virus and, in the lack of human homologs with a similar cleavage specificity, the chance of harming unintended host targets decreases, along with possible toxicity in the therapeutic use of M^pro^ inhibitors.

We observed that N3 shares similar structural motifs (Figure 1) with AT1001. These structural motifs, mainly represented by AVL residues in N3 and GVL in AT1001 (Figure 1), provided the rationale to investigate AT1001 as a potential new inhibitor of M^pro^ enzyme. The AT1001 octapeptide was docked into the catalytic cavity of M^pro^ by the peptide-specific protocol (SP-PEP) implemented in Glide (Tubert-Brohman et al., 2013) generating ~20,000 conformations. Based on docking scores (ranging from −13.535 to −7.870 kcal/mol), the best 1,000 docked poses were filtered and re-ranked by MM-GBSA protocol (Prime, 2012; Di Micco et al., 2019) and visually inspected. Our analysis suggested that AT1001 docks extremely well in the catalytic domain of M^pro^ without showing unfavorable steric interactions. The proposed binding pose presents a global turn arrangement (Supplementary Figure 3), as revealed by PROMOTIF analysis (Table 1) (Hutchinson and Thornton, 1996; Scala et al., 2017). Specifically, the octapeptide shows a β-turn (type VIa2) delimited by residues 5-8 and an inverse γ-turn formed by V3-V5.

**Table 1:** Mean values of ϕ, ψ, and χ1 angles and αC distances relative to the most representative conformers of AT1001.

**Sequence**	**i+1**			**i+2**			**αC distance** **(Å)**	
	**ϕ**	**ψ**	**χ1**	**ϕ**	**ψ**	**χ1**	**i to i+2**	**i to i+3**
V5-G8	−109.2	119.8	−177.8	−77.8	−38.2	30.0	–	4.9
V3-V5	−84.1	46.6	−60.3	–	–	–	5.8	–

**Figure 2:**
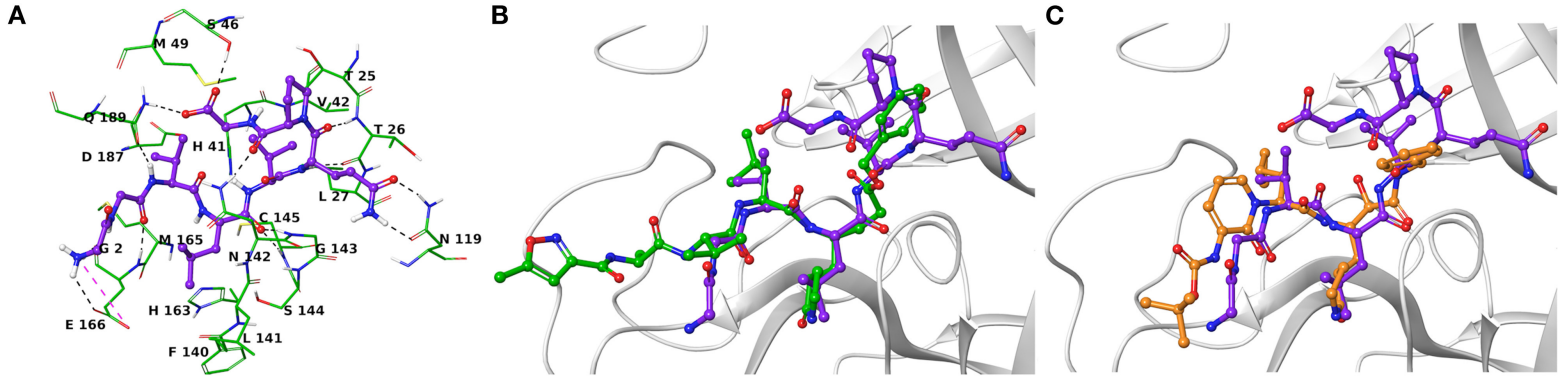
**(A)** Three-dimensional model of the AT1001-Mpro interactions. Superimposition of AT1001 with N3 **(B)** and 13b **(C)** into the catalytic site. The enzyme is represented by green tubes **(A)** and gray ribbons **(B,C)**, and is colored for the following atoms: polar H, white; N, dark-blue; O, red; S, yellow. All ligands are depicted by sticks (purple for AT1001, green for N3, orange for 13b) and balls (colored by atom type: polar H, white; O, red). The C atoms of the ligands are colored as for the sticks. The black and pink dashed lines indicate hydrogen and ionic interactions, respectively.

Another research group has independently resolved the M^pro^ crystallographic structure covalently linked to another inhibitor characterized by an α-ketoamide structure (13b, Figure 1) (Zhang et al., 2020), with crystallographic resolution similar to the N3-M^pro^ complex described above (Supplementary Figure 1). As for N3, we noticed shared structural moieties between AT1001 and 13b that prompted us to further investigate by docking simulations. The predicted binding conformation of AT1001 is superimposable onto the crystal conformations of N3 and 13b inhibitors, exploring the similar macromolecular counterparts (Figure 2).

The V3 is accommodated in a deep pocket delimited by H41, M49, M165, D187, Q189, and is H-bonded with the side chain of Q189 by its backbone NH. The L4 establishes van der Waals contacts with N142, H163, E166, F140, L141, H172, and it accepts two H-bonds from peptide NH of G143 and C145. Notably, the van der Waals interactions identified for V3 and L4 are observed in the co-crystal structure with N3, respectively, by its leucine and 3-methylpyrrolidin-2-one moiety (Figures 2B,C). Compared to N3 and 13b, AT1001 makes further van der Waals interactions with another deep sub-pocket lined up by T25, T26, L27, H41, M49 and C145. The network of H-bonds is further extended by interactions of G1 and G2 NH group of AT1001 with a backbone of E166, whereas the CO group of G2 in **2** accepts an H-bond from backbone NH of E166. The backbone NH of V3 is engaged in an H-bond with side chain CO of Q189 of the enzyme; the amide group of the Q6 side chain of AT1001 is hydrogen bonded to the side chain of N119 and backbone of T26; the backbone CO of P7 is hydrogen bonded with N142 side chain, whereas the C-terminal carboxylate is engaged in an H-bond with side chains of S46 and Q189. The N-terminal of AT1001 establishes a salt bridge with the side chain of E166 and a H-bond with the backbone CO of the same enzymatic residue. In the same article, the authors who published findings of the M^pro^ enzyme crystallographic structure bound with N3, reported Tideglusib (**4**, Figure 1) as an additional reversible inhibitor with high activity (IC_50_ = 1.55 ± 0.30 μM) (Jin et al., 2020). Even though Tideglusib is structurally different from AT1001, we also considered it in our calculations being a non-covalent inhibitor as our investigated compound. Its predicted binding energy (−55.09 kcal/mol) calculated by using the MM-GBSA method is relatively, much more inferior to that predicted for AT1001(ΔG_bind_ = −106.26 kcal/mol, Table 2). We also calculated the ΔG_bind_ of N3 and 13b, breaking the covalent bond with C145. The obtained values are higher than AT1001 (Table 2), supporting the observation that AT1001 establishes wider non-covalent interactions with macromolecular residue, respect to the co-crystallized ligands.

**Table 2:** Predicted ΔG_bind_ for **1-3** and Tideglusib *vs*. M^pro^ by using MM-GBSA of Schrödinger.

**Compound**	**ΔG_**bind**_ (kcal/mol)**
AT1001	−106.26
N3^a^	−83.85
13b^a^	−67.26
Tideglusib^b^	−55.09

a *Calculated without covalent bond to C145*.

b *Value from literature (Jin et al., 2020)*.

We analyzed the breakdown of MM-GBSA binding free energy of residues surrounding the three potential inhibitors at a distance of 5 Å (Table 3), to identify the hot spot amino acids, highly contributing to the small molecule binding. Specifically, hot spots can be defined as residues presenting a ΔG ≤ -1 kcal/mol. We observed that residues T25, T26, N119, N142, G143, C145, M165, E166, and Q189 showed values < -3.5 kcal/mol, giving a substantial contribution to the ligand recognition. It is noteworthy that these identified hot spots for AT1001 matched the residues observed for N3 and 13b and are quickly identified in the co-crystal structures with N3 and 13b.

**Table 3:** MM-GBSA Interaction Energies (kcal/mol) for residues around 5 Å from ligand.

**Residue**	**AT1001**	**N3**	**13b**
T24	−0.56	−0.51	0.01
T25	**–4.84**	**–3.49**	**–**0.70
T26	**–5.69**	**–1.11**	**–**0.95
L27	**–2.23**	**–1.41**	**−1.25**
P39	−0.22	−0.21	**–**0.11
H41	**–2.16**	**−4.71**	**−1.51**
V42	−0.75	–0.27	–0.13
C44	−0.18	–0.13	–0.13
T45	−0.13	–0.03	0.07
S46	**–2.58**	0.06	0.09
M49	**–2.64**	**−2.65**	**−1.17**
Y54	0.02	–0.07	–0.02
Y118	−0.10	–0.05	0.23
N119	**–4.12**	–0.01	–0.03
F140	**–1.13**	–0.65	**−3.12**
L141	−0.70	**−2.43**	–0.29
N142	**–11.57**	**−2.33**	**−6.81**
G143	**–3.92**	0.18	**−3.43**
S144	**–2.48**	**−1.53**	–0.99
C145	**–4.03**	**−2.50**	**−1.20**
H163	−0.95	**−2.51**	**−4.99**
H164	**–2.05**	**−1.09**	**−2.08**
M165	**–5.72**	**−8.41**	**−6.93**
E166	**–7.51**	**−5.47**	**−3.60**
L167	**–1.42**	**−2.12**	**–**0.65
P168	−0.52	**−3.03**	**−0.40**
G170	0.08	–0.20	–0.39
H172	−0.67	**−2.04**	–0.98
F181	−0.05	–0.04	–0.21
D187	−0.15	–0.66	–0.98
R188	−0.79	**−1.62**	–0.74
Q189	**–4.90**	**−7.87**	**−1.60**

*The values in bold are ≤ -1 kcal/mol*.

Based on this analysis obtained on ligand-enzyme complex, we performed an alanine scanning on AT1001 calculating the variation in the ΔG_bind_ by MM-GBSA (Table 4), to identify the crucial amino acids of **2** in the molecular recognition of target M^pro^. Specifically, we calculated the ΔΔG_bind_ referenced to the AT1001 value, and we excluded the glycine residues of **2**.

**Table 4:** Predicted ΔΔG_bind_ respect to AT1001 for alanine scanning of **2** by using MM-GBSA of Schrödinger.

**Residue**	**ΔΔG_**bind**_ (kcal/mol)**
V3A	9.53
L4A	18.86
V5A	13.42
Q6A	16.50
P7A	11.31

The analysis showed that a great contribution to the binding is given by L4, which established van der Waals contacts with M^pro^ residues. Similarly, but at lower extent then L4, V5 contributes to the complex formation. Q6 represents another notable molecular element, especially concerning its H-bond engagement with N119. Compared to residues L4-Q6, V3 and P7 give a lower contribution to the binding energy.

**Figure 3:**
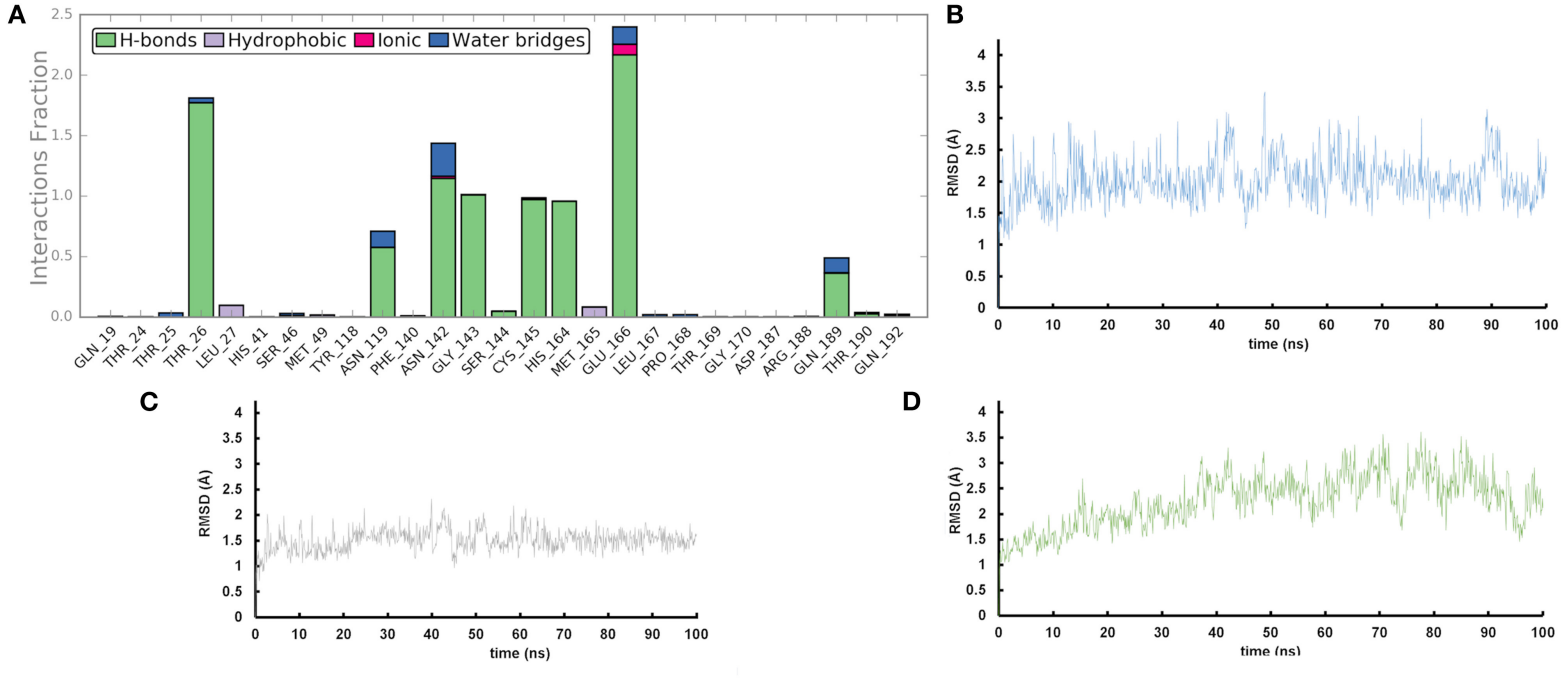
**(A)** Protein-ligand contact histograms during the simulation. **(B)** Heavy atom-positional RMSD of AT1001 respect to the protein backbone and **(C)** respect to itself (2) as a function of simulation time (ns). **(D)** RMSD of protein Cα atoms.

We performed molecular dynamics simulations (100 ns, 310 K) to monitor the time stability of the key ligand-protein interactions observed by molecular docking investigation (Zarra et al., 2009; Di Micco et al., 2014, 2019). The trajectory analysis revealed that AT1001 maintains most of the contacts with residues of the catalytic site (Figure 3A), identified from the docking conformation, during the whole trajectory (>50%) (Giordano et al., 2018b, 2019).

**Figure 4:**
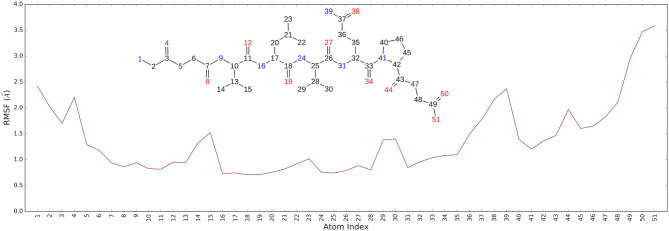
The Ligand Root Mean Square Fluctuation (L-RMSF) respect to the protein.

It is noteworthy that the residues T26, N119, N142, G143, C145, H164, E166 and N189, showing closer and more stable contacts over the simulation time (Figure 3A), was suggested as hot spots by MM-GBSA analysis of residue energies (see above). Based on these results, it is conceivable to hypothesize that these residues could be considered as hot spots responsible for the key contribution to ligand-enzyme recognition event. Specifically, tighter contacts were observed for AT1001 residues spanning from G2 to Q6, whereas, as expected, the remaining residue at N- and C-terminal positions (G1, P7 and G8) fluctuated largely (Figure 4 and Supplementary Figure 4). These observations agree with the alanine scanning analysis.

**Figure 5:**
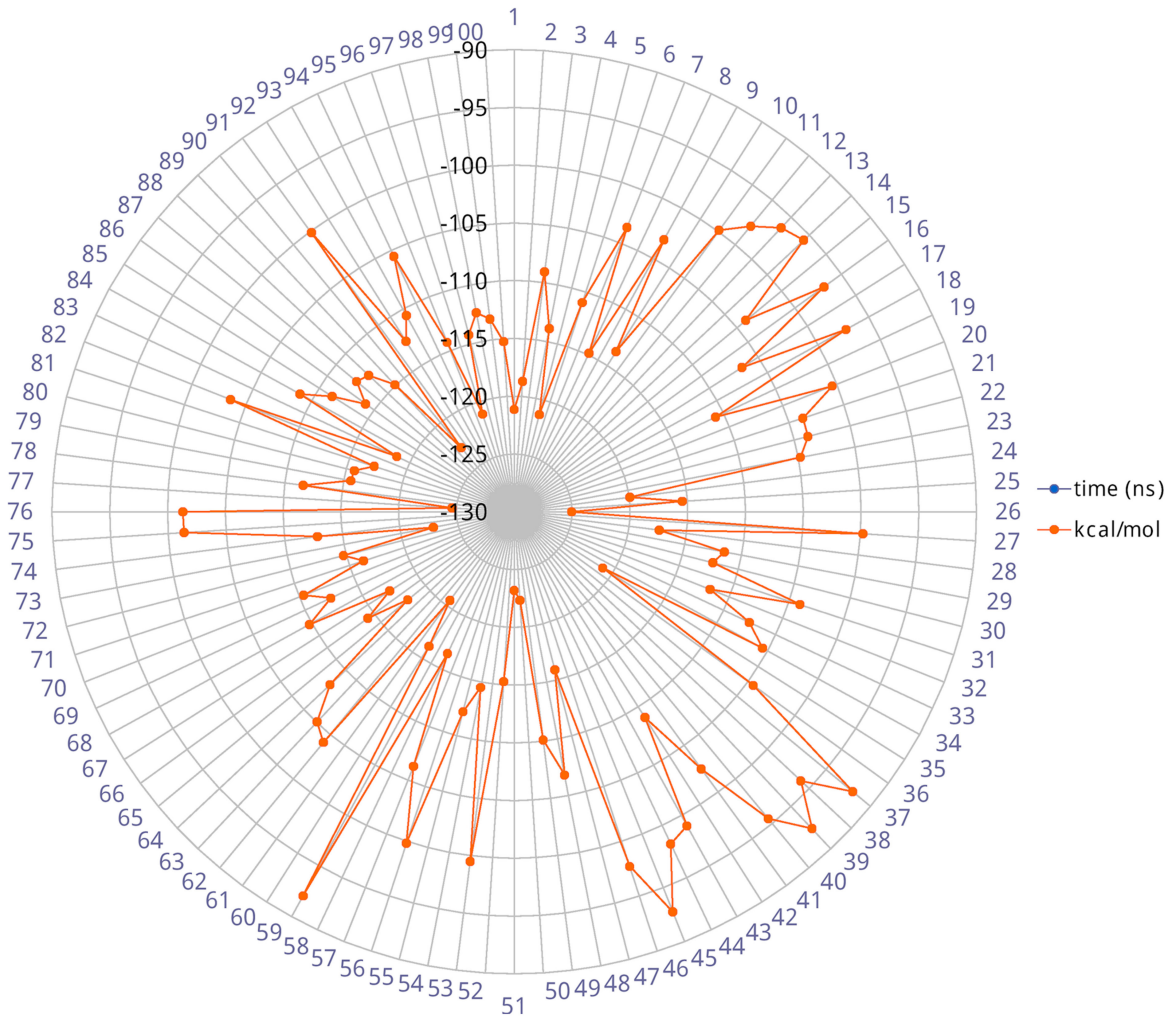
Radial plot of ΔG_bind_ predicted by MM-GBSA for 100 structures sampled each ns during the simulation.

The heavy-atom-positional RMSD (root mean square deviations) of AT1001, referenced to protein main chain, displays high constancy through the trajectory (Figure 3B), and overall, its atom-relative orientation is maintained for the period of molecular dynamics with the interacting M^pro^ counterparts (Figures 3C, 4). Furthermore, the analysis of Cα RMSD of the enzyme revealed a favorable complex line-up, without unfavorable conformational rearrangement of the macromolecule (Figure 3D). We extrapolated 100 representative conformations of the ligand-protein complex from the trajectory and we evaluated the trend of predicted ΔG_bind_ by MM-GBSA calculations (Figure 5).

The analysis revealed that the binding energies range from −125.03 to −92.0 kcal/mol, with an averaged value over time of 109.42 kcal/mol in agreement with the value obtained from docked complex. Moreover, among the calculated binding energy values extrapolated from 100 frames, only 17 predicted values were higher than the mean. Overall, the binding energy is quite conserved and the observed variations are due to the atom fluctuations and H-bond network line up/breaking over time mainly for amino acids at N- and C-terminal positions (G1, P7 and G8) of AT1001, as revealed by molecular dynamics analysis (Figures 3, 4 and Supplementary Figure 4). Combined, these structural studies suggest a potential inhibitory activity of AT1001 on the M^pro^ enzyme, laying the foundation for the development of a new generation of compounds. Specifically, the identification of proposed hot spots and key structural moieties of the ligand, highly contributing to the ligand-enzyme binding, could allow the rational design of selective M^pro^ inhibitors. The synthesis of AT1001 was accomplished by means of standard 9-fluorenylmethoxy carbonyl (Fmoc) chemical methodology through an appropriate orthogonal protection scheme (Pescina et al., 2020).

**Figure 6:**
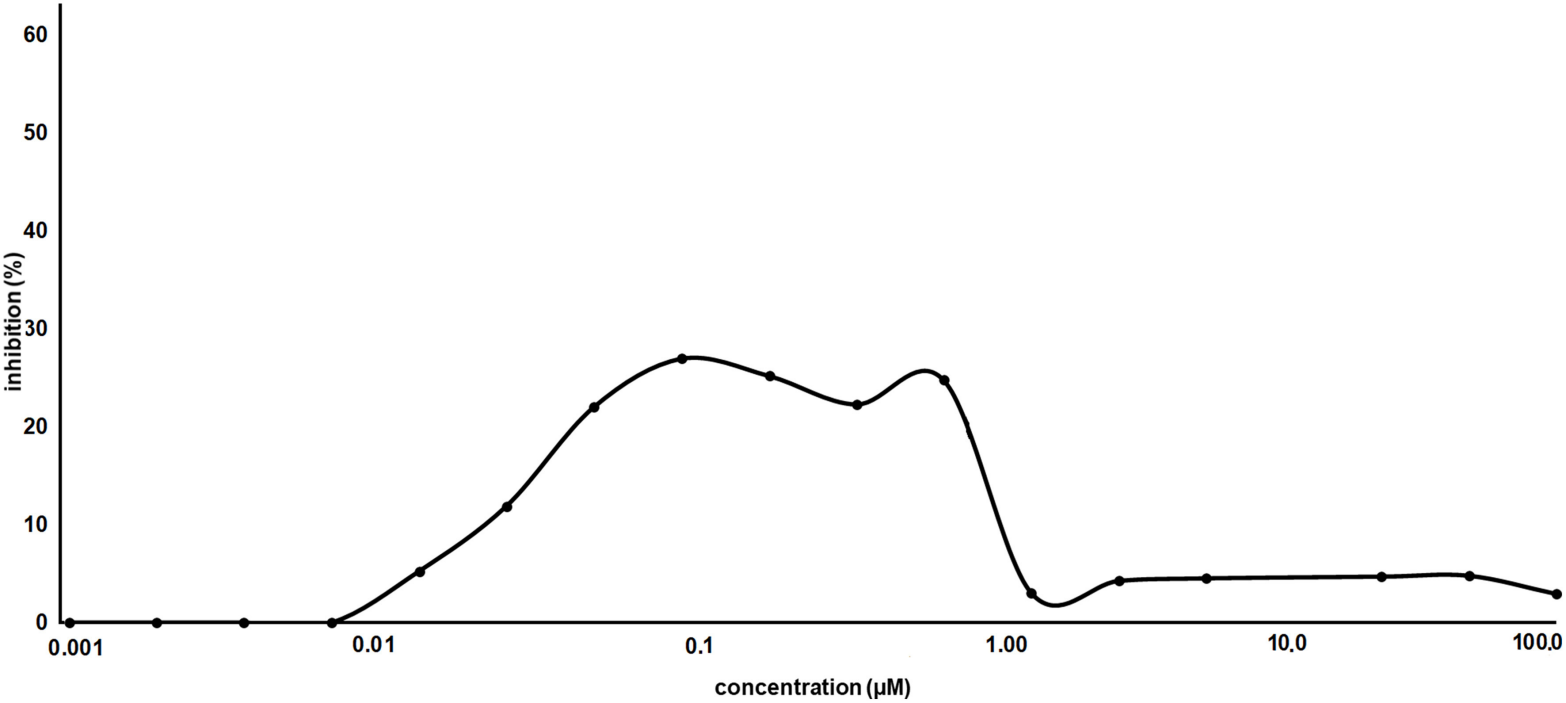
Inhibitory activity profile of AT1001 *vs*. SARS-CoV-2 M^pro^.

As proof of concept, the synthesized AT1001 was investigated for binding to M^pro^ through kinetic measurement of enzymatic activity inhibition, at different ligand concentrations, by means of FRET assay (Figure 6).

The experiments revealed that AT1001 showed a shape-bell dose-response profile with a maximum inhibitory activity at 27% (Figure 6). A starting inhibition (5%) was observed at 0.013 μM and the inhibitory activity increased until 27% at 0.1 μM. The inhibition activity was kept until 0.83 μM, followed by a drop at 3% of inhibition. According to cell-free enzymatic assay, preliminary *in vitro* studies showed that AT1001, but only at high concentrations, exerts a direct inhibition of coronavirus-induced cytopathic effect (data not shown).

## Discussion

The data we present in this study suggest that the reported protective effect of AT1001 against experimental *in vivo* murine models of ALI may be more complex than initially anticipated. Specifically, there have been two *in vivo* studies establishing the efficacy of AT1001 in mitigating ALI. The first study (Rittirsch et al., 2013) was on zonulin involvement in the ALI promotion in C57BL/6 male mice following intrapulmonary deposition of immunoglobulin G (IgG) immune complexes. The zonulin antagonist AT1001 was used and intratracheally or intravenously administrated. Moreover, a zonulin-permeating effect was stopped in the lung by means of a neutralizing antibody. In a dose-dependent manner, AT1001 (as well as zonulin- neutralizing antibodies) reduced the strength of ALI as quantitated by neutrophil accumulation, albumin leak, and proinflammatory cytokines, including IL-6 (the target of the anti-IL6 human monoclonal Ab Tocilizumab currently in trial for the treatment of COVID-19 infections). A similar result was obtained employing the bacterial lipopolysaccharide model of ALI. Through confocal microscopy on sectioned injured lungs, staining patterns for TJ proteins were discontinuous, reduced and fragmented. As expected, AT1001 normalized the TJ discontinuation and prevented the leakage of blood products into alveolus as assessed by the passing of paracellular marker dextran (both 3 and 20 kDa), and albumin. In *in vitro* and *in vivo* experimental conditions, Zonulin generated the complement fractions C3a and C5a, which was prevented by AT1001 treatment. All these findings suggested that zonulin favors the advancement of ALI both by increasing albumin leakage and complement stimulation as well as by an incremented buildup of cytokines and neutrophils, effects that were mitigated by its inhibitor AT1001, so decreasing the morbidity and mortality of the animals.

In the second study (Shirey et al., 2016), the efficacy of AT1001 therapy during a lethal influenza challenge was tested in a mouse model. WT mice were infected by influenza H1N1 strain PR8 and treated by means of vehicle (saline) or AT1001 for 5 successive days beginning on day 2 post infection. Mice treated with AT1001 showed a considerable protection and reduced clinical scores. As AT1001 diminished the pulmonary edema linked to lipopolysaccharide (LPS)- or immune complex-induced ALI (see above), in this study, AT1001 was tested to see if it mediated a reduction in lung edema triggered by PR8 infection as determined by lung wet-to-dry weight ratio. PR8-infected mice treated by sole vehicle exhibited considerably higher wet-to-dry ratios than the group treated by AT1001, suggesting that the protective effect of the drug during influenza infection consists in ALI attenuation by diminishing pulmonary edema. In both studies, AT1001 administered either systemically (IV) or locally (mucosal airways) showed a strong safety profile, with no death or other complications reported.

With the present study, we hypothesize that, along with its proven protective effect on increased mucosal and vascular permeability in the airways typical of patients affected by ALI or ARDS, AT1001 may also exert a direct anti-SARS-CoV-2-specific effect by blocking its M^pro^ enzymatic activity. Coronaviruses have a single-stranded, 5′-capped, positive RNA polymer, ranging from 26 to 32 kb and encompassing at least 6 open reading frames (ORFs). The ORF1a/b covers nearly two-thirds of the genome and encodes replicase proteins (Perlman and Netland, 2009). The translation starts in ORF1a, then continuing in ORF1b following a −1 frameshift signal. Both ORF1a and ORF1ab, also, respectively, mentioned as pp1a and pp1ab, are mainly processed by the viral encoded M^pro^. Therefore, M^pro^ represents a limiting step for virion replication, making it an attractive target to design anti-SARS-CoV-2 drugs. Our findings show that AT1001 potentially binds to the M^pro^ catalytic domain, and that the observed intermolecular interactions are kept over time. These theoretical observations match the reported structure-activity relationship in literature for the known inhibitors, which would place the AT1001 inhibitor above them in terms of potential efficacy. We experimentally proved a valuable binding toward M^pro^ by AT1001, acting already at 0.013 μM. Interestingly, we observed a bell-shaped dose-response curve, probably due to enzymatic hydrolysis on AT1001 as a peptide. This specific experimental behavior does not allow a direct comparison with reference compounds, despite the theoretical predictions could suggest a tighter binding. Overall, it is quite hard to show the inhibitory effect by non-covalent inhibitors and the obtainment of non-standard sigmoidal dose-response curve has been already reported, such as for falcipain-2 binders (Alberca et al., 2019).

Contrary to other possible M^pro^ inhibitors recently reported in the literature (Jin et al., 2020; Zhang et al., 2020), AT1001 has been amply tested both in animal models and in humans showing very strong safety and efficacy profiles when administered either systemically or mucosally for local surface therapeutic activity.

Concerning human studies, AT1001 (with the name Larazotide acetate) has been studied in seven clinical trials (three Phase 1 [two in healthy adults and one in adults with celiac disease] and four Phase 2 studies) (Leffler et al., 2012, 2015; Kelly et al., 2013). A Phase 3 study (NCT03569007) in adults for the relief of celiac disease symptoms is ongoing. As noted earlier, results from these studies (involving more than 800 subjects), including the Phase 3 study to date, show a good safety profile with an absence of serious or severe adverse events (Varga et al., 2020).

The reported pediatric multisystem inflammatory syndrome and the co-morbidity in SARS-CoV-2-infected adult patients involving cardiovascular, renal and central nervous system districts secondary to endothelial barrier dysfunction—with subsequent complement activation, cytokine storm, and generalized thrombosis—adds another strong rationale for the use of AT1001, not only to treat the primary pulmonary district and reduce viral load due to its direct anti-viral activity, but also to protect other organs by favoring endothelial barrier competency and reducing the viral load by blocking its replication.

SARS-CoV-2 is a coronavirus that initially infected the human host late in 2019 with devastating effects around the globe. We currently have no specific therapeutic options against SARS-CoV-2, but instead are repurposing drugs as experiments in clinical trials. Based on the results presented in this study and the already reported protective effect of AT1001 against zonulin-mediated pulmonary damage typical of viral infections, we propose that AT1001 may represent an innovative therapeutic option to multi-target both early COVID-19 upper airway infections (reducing interpersonal spreading) and distal airway infections responsible for the functional failure of lungs and other organs.

## Data Availability Statement

The original contributions presented in the study are included in the article/Supplementary Materials, further inquiries can be directed to the corresponding author/s.

## Author Contributions

SDM and AF: conceptualization, writing—original draft preparation, supervision, and project administration. SDM: methodology, validation, investigation, and visualization. MCS: synthesis. SDM and MS: formal analysis. GB: resources. SDM and SM: data curation. SDM, PC, and AF: writing—review and editing. AF: funding acquisition. All authors have read and agreed to the published version of the manuscript.

## Conflict of Interest

The authors declare that the research was conducted in the absence of any commercial or financial relationships that could be construed as a potential conflict of interest. The handling Editor declared a past co-authorship with the authors MCS, MS, and PC.

## Supplementary Material

**Supplementary Material includes**: Ramachandran plot of docked pose of AT1001; The ligand torsions plot of every rotatable bond in the ligand throughout the simulation trajectory (100 ns); Analytical HPLC trace; ESI-MS spectrum.
