# Supplementary material for: In silico Analysis Revealed Potential Anti-SARS-CoV-2 Main Protease Activity by the Zonulin Inhibitor Larazotide Acetate
Source: Front Chem. 2021 Jan 15;8:628609. doi: 10.3389/fchem.2020.628609 (PMC7843458; doi:10.3389/fchem.2020.628609)
Supplement: Supplementary file 1 [file Table_1.DOCX]

Supplementary Material

In Silico Analysis Revealed Potential Anti-SARS-CoV-2 Main Protease Activity by the Zonulin Inhibitor Larazotide

Simone Di Micco^1^, Simona Musella^1^, Maria C. Scala^2^, Marina Sala^2^, Pietro Campiglia^1,2^, Giuseppe Bifulco^2^, Alessio Fasano^1,3*^

^1^European Biomedical Research Institute of Salerno (EBRIS), Salerno, Italy

^2^Department of Pharmacy, University of Salerno, Fisciano, Salerno, Italy

^3^Mucosal Immunology and Biology Research Center, Massachusetts General Hospital–Harvard Medical School, Boston, Massachusetts, USA

*** Correspondence:**Alessio Fasano
afasano@mgh.harvard.edu

**Figure S1.** Ramachandran plot of docked pose of AT1001 calculated by PROCHECK by using a hypothetical resolution of 2 Ǻ. The color code is: white, disallowed region; cream, generous region; yellow, allowed region; red, most favourable region. Black markers indicate amino acids in favourable regions.

**Figure S2.** The ligand torsions plot of every rotatable bond (RB) in the ligand throughout the simulation trajectory (100 ns). The top panel shows the 2d schematic of a ligand with color-coded rotatable bonds. Each rotatable bond torsion is accompanied by a dial plot and bar plots of the same color. Dial (or radial) plots describe the conformation of the torsion throughout the course of the simulation. The beginning of the simulation is in the center of the radial plot and the time evolution is plotted radially outwards. The bar plots summarize the data on the dial plots, by showing the probability density of the torsion.

0,0

0,5

1,0

1,5

2,0

2,5

3,0

3,5

4,0

4,5

5,0

5,5

6,0

6,5

7,0

7,5

min

0

25

50

75

100

125

150

175

mV

lAT1001.lcd Detector A 220nm

**Figure 3.** Analytical HPLC trace of Larazotide at 220 nm.

**Figure 4.** ESI-MS spectrum of AT1001. FTMS + p ESI Full ms [150.00 - 2000.00].
